# Protecting antiquities in early modern Rome: the papal edicts as paradigms for the heritage safeguard in Europe

**DOI:** 10.12688/openreseurope.13539.1

**Published:** 2021-05-13

**Authors:** Chiara Mannoni

**Affiliations:** 1 Ca' Foscari University of Venice, Venice, Italy

**Keywords:** Heritage protection, heritage legislation, early modern centuries, Rome, art market, excavations, export, antiquity.

## Abstract

The edicts issued in Rome between the fifteenth and the eighteenth centuries are the earliest legislation conceived for the preservation and supervision of heritage in Europe. Not only did such regulations aim to protect monuments, antiquities, and – at a later stage – paintings from the risks of damage and deterioration, but also established a legal framework against their illegal exportation and excavation.

In this study the gradual development of this vast corpus of legislation is considered within the variations of artistic scholarship, legal knowledge, artistic taste, and the art market in Europe between 1400s and 1700s. The mutual implications of juridical constructs and practices of supervision are evaluated together with interdisciplinary factors – such as the rise of collections and museums – to shed light on the development of the concepts of ‘heritage protection’ in early modern Rome. Specific analysis will also involve the gradual expansion of the definition of ‘antiquity’ and ‘artefact’ in papal legislation, as well as the establishment of innovative instruments to prevent and circumvent misdemeanours.

One final consideration is given to the launch of local procedures of heritage protection in other states in Europe. Considering the cultural and historical backgrounds of each individual place, this study will demonstrate that the idea of safeguarding what was thought of as ‘collective heritage’ emerged consistently in eighteenth-century Europe following the paradigms of the papal edicts.

## Introduction

There can be no effective safeguard of historic and artistic heritage without the recognition of what it is that needs to be protected – and which methods are suitable for its achievement ^1^. Conservation results from the identification of an object as an artwork and can be explained as the tangible consequence of a mutual awareness of what art is and what it is not. Similarly, the legal and administrative practices that are applied to put protection into effect are strictly related to the perceptions and systems of values of each epoch and community. From a historical perspective, it can be observed that the acknowledgment of the qualities that make an object worthy of safeguard does not always coincide with the moment of its physical creation. This means that, although artefacts and artistic goods have always existed, the belief that they constitute ‘heritage’ requiring rules of maintenance, management, and care, have been generally established at later stages. In this respect, Emiliani has claimed that “we cannot assume that the epochs of creativity have developed at the same time a deep historic reasoning, able to produce both the artistic categories and the terminologies to approach them” ^2^.

The current concepts of ‘protection’, ‘conservation’, and ‘custody’ of the material products of the past, as well as the idea that these are part of ‘collective heritage’, started to develop, or even had their origin, in the early modern Papal States. It is not surprising that the cultural mindset capable of conceiving the first models of safeguard of older artefacts arose in the Italian peninsula just before the Renaissance. In Italy, the relics of the ancient Roman Empire were disseminated and prominent all over its territory, and they had been languishing in a state of neglect since the beginning of the Middle Ages. In particular, the Catholic church elected the city of Rome as the official abode of the popes, gradually embracing the purpose to gain symbolic control over ancient Roman monuments to enhance its political and religious power. By the early modern era Humanism was already flourishing, and the artistic and cultural trends of the Renaissance were developing. Antiquity was adopted as a prototype and ideal of excellence in literature and the visual arts, both in the Italian peninsula and other areas of Europe. Within this classically inspired background, the Catholic popes began referring to ancient Rome in their religious agenda for the reconstruction of a new Rome, also promoting the publication of the first regulations to protect antiquities and monuments from destruction, improper use, and liming of marbles.

The Papal States issued the first law on the protection of antiquity as early as 1425, introducing more than 30 further directives in the following four centuries ^3^. The *corpus* of this legislation not only formed a legal tradition itself, legitimising both the safeguard of the ‘past glories’ of Rome and the popes’ right to pursue their agenda on them, but also established a juridical model to launch new procedures of heritage protection in other places in Europe. As will be clarified later in this article, there were cultural, historical, and methodological variations in the formation of internal laws in different states and cities, including rules dedicated to the preservation of specific classes of monuments. In wider terms, however, the idea of ‘juridically’ protecting what was thought of as collective heritage emerged quite consistently in several European countries only in the 18 ^th^ century, following – in fact – the centuries-old models of the Papal States.

## The fifteenth century

In 1425, the edict *Etsi in Cunctarum* of Pope Martin V landmarked the beginning of the popes’ concerns on the preservation of what was regarded as heritage, intended at this stage as any site that had a “civil” significance for the community, such as “alleys, streets, and squares” ^4^. It is noteworthy that Martin V had studied both arts and canon law at the University of Perugia before pursuing his ecclesiastic career ^5^; such a background was certainly crucial in the construction of his later understanding of humanities and classicism in connection to legislation. Regarding the contents of the edict, both “public and private places” were included under protection, together with “buildings made of stone, wood, beams, tables, [as well as] roofs, floors, storages and bridges, gates, passages, watercourses, canals and drains, and also urban and rustic farms, fields, [and] gardens” ^6^. These structures were guarded against destruction, illegal sale, occupation, and improper use. To achieve his purpose, the pope established the office of *Magistri Viarum* – the road master – who was responsible to invigilate on these properties and ensure their adequate conservation within the city. Even though the edict did not explicitly mention antiquities and monuments, it stated the need to oversee the decorum of longstanding and centuries-old goods, both private and public, alongside any other community asset.

The following edict *Cum Almam Nostram Urbem*, issued by Pope Pius II in 1462, explicitly included ancient remains and monuments under protection, together with “Basilicas, Churches, religious and holy grounds, which contain Saints’ shrines and reliquaries” ^7^. Antiquities, at this stage, were fully accepted as models of virtue and glory in the Catholic Church; for this reason, the pope prohibited their “demolition, destruction, shattering, or breakage”, including the liming of marbles and their reuse as building materials ^8^. Nevertheless, specific permissions to remove ancient remains could be granted by the pope himself within bulls or apostolic briefs. Indeed, all the earlier legislation issued in Rome reflected such an evident dichotomy in the attitude towards antiquities: while promoting, on the one hand, their preservation for their symbolic significance, the popes retained the right to recommend their reuse for specific purposes. In 1474, the edict *Cum Provida* of Pope Sixtus IV presented again this duality. It condemned the despoliation of “scarlet and red marbles, along with other stones of different quality and colour”, in this case denoting merely the materials that had already been reused for constructing new churches in recent years ^9^. In 1515, Pope Leo X ordered the appointment of the artist Raphael Sanzio as *Praefectus marmorum et lapidum* – prefect for marbles and stones – in Saint Peters, entrusting him to supervise the adequate protection of “ancient marbles and engraved stones”, as they were representative monuments of the past ^10^. Yet, the artist was also asked to grant permissions to cut, saw, and reuse ancient materials to build the new basilica in Rome ^11^. It is clear that, at this early stage, the papal legislation on antiquity protection was neither systematic nor consistent.

## The sixteenth century

A first change of approach in the practices of heritage safeguard occurred in the sixteenth century, when concerns regarding the loss of movable items arose in connection to the development of an embryonic art market. In fact, in the previous century the popes’ attention had exclusively focused on the state of preservation of immovable buildings, as well as the risks endangering the conservation of monuments, temples, inscriptions and ornaments – that is, works that were either fixed to a structure or too big to move. As soon as the demand for antiquities increased, following the escalation of dynastic collections and the so-called *Wuderkammern*, the dealing in small items, curiosities, memorabilia, and sculpted fragments increased all over Europe. The popes rapidly understood that the absence of control on these portable works could compromise the rich endowments of Rome, and the Catholic Church itself. In 1534, in his edict Pope Paul III attacked the “disgraceful” relocations of “statues, emblems, marble and bronze slabs, as well as scarlet, Numidian and other coloured stones” from Rome to “foreign lands and cities” ^12^. He created the first ever post of commissary of antiquity to control the exports of movable objects and regulate the improper use and destruction of immovable buildings, appointing Latino Giovenale Manetti ^13^. A few years later, in 1562, Pope Paul IV divided the post into an *Antiquitatem Praefecti* and a *Conservatores* – a prefect and a conservator for antiquities – pursuing to improve control on the outflow of ancient works ^14^. The loss on these items, evidently, was increasing in spite of any regulation issued to deter it. Paul IV’s edict also widened the categories of objects to protect. “Ancient marbles, both raw and worked, statues, busts, metals, precious stones, coins, vases, and cups made of bronze, silver, and gold, as well as slabs and inscriptions” ^15^ were included to its supervision, in the attempt to retain, specifically, the varieties of goods which were sought for the *Wunderkammern* and collections in Europe. Interestingly, the edict also affirmed the pope’s intention to block the production and trading of forged antiquities, “which [were] sold as ancient works” and ultimately harmed the honour and good reputation of Rome ^16^.

Following this interpretation, it is also worth mentioning the constitution *Quae Publice Utilia*, issued by Pope Gregory XIII in 1574 ^17^. Although this bill was not dedicated specifically to antiquities and monuments, it affirmed the superior interest of the state and the community – rather than the private individuals – in the use of the assets of Rome ^18^. This was one of the earliest and firmest cultural positions in this regard in Europe and would have profound implications on the development of the concept of ‘public heritage’ in recent attitudes to its conservation and legal protection.

##  Linguistic aspects

Before analysing the edicts issued in the seventeenth-century, we should consider the aspects in papal legislation which can be defined as structural and syntactic, mostly concerning its linguistic attributes. The laws issued in fifteenth- and sixteenth-century Rome were written in Latin – to be more specific, a refashioned early modern Latin which was the product of the Renaissance humanists’ efforts to study Latin in ancient sources. The process of rediscovery of this ancient language was not smooth at that time: it should be considered that nearly 1,000 years had elapsed between the fifth century CE, when the Western Roman Empire finally fell, and the fifteenth century, when scholars needed to revitalise classicism and ancient culture pursuing a philological approach. Indeed, as compared with the Roman Empire, new concepts and ideas had matured in the 1400s, requiring new forms of expression as different systems of thinking and reasoning had developed both within medieval and renaissance scholarship. Furthermore, language and society had changed, requiring innovative terminologies, categories, lexica, and semantics within communication. In short, fifteenth-century Latin was not the classical idiom of Cicero and Virgil, but a completely new Renaissance Latin ^19^.

Besides these morphological aspects, the implications of using Latin in legislation should be considered in relation to the wider awareness of the need to protect the heritage within society. Latin – Renaissance Latin – was the official language of the Catholic Church, and the entire *Corpus iuris civilis* and *Corpus iuris canonici* were constructed on it ^20^. However, it is problematic to affirm how much the edicts on the protection of antiquities were effective and respected by the local communities in the Papal States. Most likely, common people were not able to understand and follow their legal prescriptions, as they mostly spoke vernacular and early modern Italian. Although this certainly also applied to other spiritual and secular rules imposed by the popes, it should be observed that there was only one commissary of antiquity in Rome, as compared with other sectors of the papal administration. It was evidently impossible for a single man to manage and control the extensive amount of antiquities in the city. As a consequence of both the linguistic issues and administrative shortcomings, the laws had generally remained a nonconcrete list of stipulations, which were ineffectual and impossible to apply. At this stage, therefore, legislation on heritage protection represented a declaration of intent lacking any successful tool of implementation, in spite of the significance of its cultural and conceptual elaborations.

## The seventeenth century

After the short lifespan of the 1562 positions of prefect and conservator for antiquity – which *de facto* were never operational – subsequent edicts reverted to the model of a sole commissary. Between the sixteenth and late eighteenth centuries, sixteen commissaries would be documented in the records on the antiquity safeguard in Rome ^21^. Such an arrangement, however, was clearly inadequate to control the city’s wealthy assets. In the seventeenth century, the popes experimented new procedures to make the law effective: firstly, they entrusted the cardinal camerlengo – the state chamberlain and treasurer – to compile regulations and deal with the heritage management; secondly, they established severe punishments and fines for those who ignored or disregarded the law. The latter aspect in particular, marked a clear change in the attitudes towards the heritage protection; from this moment on, misappropriations and smuggling of artefacts were no longer perceived as issues of personal and religious consciousness, but tackled as civil misdemeanours ^22^.

In 1624, the so-called *Edict Aldobrandini* by Cardinal Ippolito Aldobrandini extended protection to modern artefacts in addition to ancient items. Major concerns at this stage involved not the destruction and despoliation of antiquities, but their illegal excavation and unapproved sale. Surveillance, therefore, encompassed mainly the digging and export of “Figures, Statues, antiquities, Ornaments, works both ancient and modern, made of marble, metal, or stone of any kind, both integer and in pieces” ^23^. It is significant that not only was this edict written in early modern Italian, but it also prescribed a list of punishments to apply in the cases of infringement, consisting of confiscations of artefacts, loss of employment, fines of up to 500 crowns, and corporal punishments, such as the strappado ^24^.

A few years later, in 1646, the *Edict Sforza* by Cardinal Federico Sforza made the application of the punishments more rigorous, also providing a new sophisticated list of items to incorporate under protection: “statues, figures, bas-reliefs, columns, vases, alabasters, agates, jaspers, amethysts, and other marbles, precious stones, worked and uncut stones, busts, heads, fragments, pillars, pedestals, inscriptions, [as well as] other ornaments, friezes, medals, cameos, or carvings on any stone, metal, gold, silver, of any ancient or modern material, and also figures, ancient paintings, or other works sculpted, painted, carved, assembled, carved […]” ^25^. The edict was mostly based on an antiquarian predilection, in which the category ‘ancient paintings’ referred to frescoes, encaustics, and murals typical of ancient Roman and early Christian art. Overall, the body of the law appeared very long and repetitive, reinstating the lists of objects and the punishments for any infringement recorded. This makes it easy to affirm that illegal excavations, smuggling, alterations, and destruction of antiquities were still common habits in Rome at this time, increasing regardless of any innovation introduced to deter them. Further new edicts were published just a few years later. The *Edict Altieri* of 1686 and the first *Edict Spinola* of 1701, by Cardinals Paluzzo Altieri and Giovan Battista Spinola respectively ^26^, replicated the same lists of objects and the punishments from the previous *Edict Sforza.* Even so, despite the reinstatement of the impositions, the situation did not improve. Evidently, the losses of artefacts were still significant, and the procedures of control insufficient.

## The early eighteenth century

To follow the subsequent development of a consistent concept of heritage, as well as its related practices of protection, we need to acknowledge the innovations that the Enlightenment introduced within culture, philosophy, juridical knowledge, and – more so – the art system throughout the eighteenth century. Although the Papal States has been described commonly as a decayed isolated place, particularly after the Baroque’s extravagances in the mid-1600s ^27^, Rome was designated as the major destination for the Grand Tour in 1700s and the hub of the antiquarian market in Europe. Its connections with the other European states, therefore, became multifaceted and extremely vivacious in this period ^28^. Rome attracted the interests of both scholars engaged in cultural exchanges within the so-called Republic of Letters ^29^, and international dealers, aristocrats, and artists seeking treasures to increase their homeland collections ^30^. In this scenario, it is clear that while the popes, on the one hand, wished to foster the city’s good reputation and influence, on the other hand they supported the definition of improved systems of heritage protection. The latest developments in artistic and legal knowledge, as well as the ever-growing outflows of artefacts towards Europe, accelerated the establishment of innovative solutions in this regard.

In order to manage the large quantities of antiquities discovered in excavations, the second *Edict Spinola* of 1704 imposed that any new item found in Rome be declared to the office of camerlengo without delay ^31^. A drawing of the artefacts that could not be preserved due to the diggings was also required for documentation. Nevertheless, the foremost innovation of the edict was in the incorporation of books and documents under protection. “Manuscripts in Vernacular, Latin, Greek, or Hebrew […] divided, torn, or loose, as well as Tools, Proceedings, Inventories, Letters, Bulls, Briefs, Charters and any other sort of paper” were banned from sale and improper use in Rome for the first time ^32^. This new, fundamental category of heritage would be given a specific form of safeguard in 1712, within the third *Edict Spinola* ^33^.

It is interesting that the fourth – and final – *Edict Spinola* of 1717 specifically addressed “all foreigners and other Prince’s subjects […] who have resided in Rome for at least one month”, tackling the problem of travellers and grand tourists involved in the trading of antiquities, both legal and illegal ^34^. Although the edict did not add any significant element to the concept of heritage, it aimed to regulate and, possibly, limit the exports of artefacts to England, Spain, France, and the German area in particular ^35^. Accountability regarding the relocation of artefacts was also enforced on the owners of inns, taverns, hostels, and guesthouses – that is, the places where foreigners used to spend time. These categories of professionals were asked to affix a copy of the edict for their guests to read. A few years later in 1726, the first *Edict Albani* by Cardinal Annibale Albani brought attention back to the excavation of antiquities ^36^. Evidently, illegal quarrying and smuggling were perceived as two different categories of misdemeanour at this stage. The edict declared as illegal any unapproved digging of “Statues, or their fragments, Busts, Heads, Bas-reliefs, Pedestals, Columns, Capitals, Inscriptions, Vases, Urns, and other ancient ornaments”, including their demolition and alteration ^37^. Nevertheless, it is clear that illegal excavations and exports of artefacts were the two faces of the same medal: they could not be addressed and resolved disjointedly.

The subsequent refinement of the definition of heritage and its practices of protection can be observed in the second *Edict Albani* of 1733 ^38^. This new regulation, at the outset, reissued the sixteenth-century prohibition on the trade of “things [...] altered and counterfeit” ^39^, recognising them as antiquities and small objects which were not only falsifications of originals but were also sold at disproportionate prices to foreigners. Most importantly, the edict introduced the protection of “Paintings, Mosaics, and Pictures […] both ancient and modern” ^40^, assigning value to movable paintings after a three-century-long exclusive interest in antiquities in Rome. Clearly, these conceptual elaborations cannot be explained by omitting the role of the wider European art market and scholarship of that time. Indeed, the trading of fake antiquities had inflated in Rome during the last decades of the 1600s, caused both by the growing demand for artworks from European collectors and a serious economic crisis involving the entire Papal States ^41^. In order to meet the requests of the market, impoverished artists started counterfeiting and selling fake antiquities, seeking to increase their income. In addition to this, increasing exports of paintings were connected to the growth of interest in Italian Renaissance art by collectors and scholars in Europe. Paintings, moreover, were extremely easy to export from the Papal States, as they had been omitted from the systems of surveillance in the previous centuries ^42^. Therefore in 1733, wooden and canvas paintings were finally included in the law, responding to the new trends within scholarship and the art market ^43^.

##  The late eighteenth century

The culmination of early modern papal legislation on the heritage safeguard has been mostly associated with the issuing of the *Edict Valenti Gonzaga* by Cardinal Silvio Valenti Gonzaga in 1750 ^44^. This can be regarded as the most advanced result of the papal efforts on the protection of antiquities and artefacts up to that time, remaining operational for the subsequent 50 years ^45^. Interestingly, this was also the first regulation that not only had effective outcomes on the administration of the heritage in Rome, but also approached the control on the excavations and that on the exports as an interconnected issue ^46^.

In the first half of the1700s, massive campaigns of digging and surveying were started in different areas of the Italian peninsula and central Europe ^47^. In particular, Herculaneum was discovered and unearthed in the Kingdom of Naples in 1738 and Pompeii also followed ten years later. The rigid protectionism of the Bourbons in the management of both sites, comprising restrictions in the publications of the new findings as well as discouragement of visits in the areas, might have stimulated the pope to pursue stricter policies on the control of excavations and sales of artefacts in Rome ^48^. Besides this, it should be considered that the half of the eighteenth century reflected a change of approach within the juridical disciplines altogether. Philosophers such as Montesquieu, Voltaire, Rousseau, and Beccaria were redefining rights and duties of both citizens and monarchs in modern societies, while several European countries reformed their domestic juridical systems following the principles of the legal Enlightenment ^49^. In the Papal States, Pope Benedict XIV initiated a season of reforms soon after being elected in 1740. The entire ecclesiastic arrangement was renovated during his 18-year-long pontificate. Changes were made to the trading system, public offices within the curia, administrative and judiciary assets, as well as the theological and clerical schemes ^50^. Benedict XIV was particularly captivated by the theoretical reformations of the scholar Ludovico Antonio Muratori in the field of jurisprudence, described in the volume *Dei difetti della giurisprudenza* of 1742, which indeed was dedicated to the pope in memory of their friendship ^51^. Although Muratori’s project to compile a new civil code – if we can use this contemporary term – was not achieved, the pope was clearly persuaded by his recommendations to remove limits of implementation and contradictions in law, possibly offering him a motivation to support new policies and approaches for the heritage protection.

Regarding the contents, three major novelties were established within the edict of 1750. The first concerned the concept of heritage, which – at this time – consisted of: “Statues, Figures, Bas-reliefs, Columns, Vases, Alabasters, Agates, Jaspers, Amethysts, and other precious Marbles, Jewels, worked Stones, Busts, Heads, Fragments, Small Items, Pedestals, Inscriptions, or other Ornaments, Friezes, Medals, Cameos, Gems, Coins, or carvings in any Stone, or Metal, Gold, Silver of any ancient or modern material, and also Figures, Paintings, Ancient Paintings, or other works sculpted in any kind of material, painted, carved, assembled, worked, or made in any way […] which are in Rome and out of Rome” ^52^. Even though the list was still based on the centuries-old idea of artistic miscellanea, the inclusion of the open-ended clause was intended to suggest objects that were not explicitly mentioned in the provisions of the edict. Such an opening towards further unrecorded items might denote that the definition of ‘artefact to protect’ had begun to be understood as a concept in continuous development, impossible to define *a priori*, but to be established now and again according to each case. This also included a very early allusion to the works that were placed outside of Rome, that is, in the provinces of the state.

A further innovation related to the Capitoline Museum in Rome. Prescribing that any item confiscated due to misdemeanours should be transferred to the city gallery, the edict encouraged, at least indirectly, the right of the community to access and enjoy art ^53^. In this perspective, it is significant that the foundation of the Capitoline Museum in 1734 and the later establishment of the Vatican Pius-Clementine gallery in 1770 responded specifically to the intention of retaining the finest local artefacts within the state, by controlling both their excavations and sales. The opening of these prominent galleries substantiated the papal solution to contain the massive outflow of artefacts in the art market, as well as the illegal removals of private aristocratic collections – such as the Odescalchi, Chigi, and Giustiniani – that had been increasing in those years ^54^. The edict offered a clear, political position in this regard: not only did it reaffirm the rights of the state over those of private individuals in the use of heritage – as already insinuated in the bill of 1574 – but it also introduced the idea of the public destination and meaning of art in societies.

The final, and most relevant, reform concerned the establishment of a body of a main commissary and three assessors to carry out the procedures of control and safekeeping of the heritage in Rome. As already mentioned, the most significant shortcoming of earlier papal regulations was that they did not establish a thorough system of supervision across the city. Seeking to improve the procedures of inspection on both excavations and sales, three new positions of assessor were expected to supplement the capacity of the main commissary, taking care respectively of “Paintings”, “Sculptures”, “Cameos, Medals, Engravings, and any other kinds of Antiquity” ^55^. The position of commissary, for its part, started to be filled with extremely acknowledged appointees, such as Johann Joachim Winckelmann (1763–1768) and Filippo Aurelio Visconti (1785–1799) soon after 1750 ^56^. The former deputy, in particular, would have an enormous impact on the systematisation and understanding of the history of ancient art in Rome and the whole of Europe as well. Winckelmann would initiate the interpretation of antiquity into distinct styles, typologies, and iconographical series corresponding to specific historical chronologies and epochs, as he recorded in his major volumes *Geschichte der Kunst des Alterthums* of 1764 and *Monumenti antichi inediti* of 1767 ^57^. The gradual codification of art history and its modern approaches, in this regard, not only complemented the establishment of public museums and galleries, but also concurred with the elaboration of more advanced juridical constructs on the heritage protection. Surely, it can be affirmed that the new administrative system set up to supervise, maintain, and defend the treasures of Rome ultimately functioned thanks to the emerging of such an innovative theoretical and methodological foundation.

##  Legal aspects

To be valid and fully operative, legislation on the safeguard of heritage requires a clear definition of the items to which the rules are to be applied, together with a body of executives allocated for the implementation of the prescribed instructions. A further essential element is a set of legal instruments to be used in the procedures of management and control. The evolution of papal legislation between 1425 and 1750 – described in the previous paragraphs – reflected the gradual development of concepts, administrative systems, and tools devised to preserve the artefacts within the state. To give an example, after the impasse of the earlier edicts of the 1400s and 1500s, the *Edict Sforza* of 1646 proposed an early practice of right of first refusal to minimise the losses of antiquities coming from excavations ^58^. According to this procedure, no one granted with a licence to dig in Rome could sell the new findings prior to the commissary’s inspection and estimation. Indeed, a suitable portion of these finds was to be allotted to the Apostolic Chamber, which was also entitled to confiscate all the pieces in case of infringements. In 1750, the *Edict Valenti Gonzaga* extended this system to the art market too, assigning the artefacts obtained here to the papal museums ^59^. In a wider perspective, it can be observed that legislation – at least at its onset – was largely constructed on castigatory procedures, such as confiscations, corporal punishments, loss of employment, and monetary charges. This implies that in the early stages, there was no instrument for preventing or circumventing the risks of smuggling and destruction of artefacts.

The procedure to grant – or reject – a licence can be interpreted within this background as well. The early introduction of the need to procure such a permit emerged in 1534, concurring with the creation of the post of commissary of antiquity and involving both excavations and exports ^60^. Although, initially, the approval of the permits remained in the hands of the pope, the new commissary was appointed to inspect the quarries, control the relocations of artefacts, and oversee the maintenance of the monuments in Rome. The *Edict Sforza* of 1646 also conferred him the faculty to endorse or veto licences – which at this time needed to be validated by the camerlengo – after “determining the items’ quality and quantity” ^61^, that is after having inspected both the works proposed for export and the new findings coming from excavations. In 1717, the fourth *Edict Spinola* assigned him extra supervision on any trade of artefacts within the state, including those that did not entail their export abroad, with the aim to strengthen control on the circulation of antiquities in Rome ^62^. As can be observed, within this set of evolving rules the essential parameters to regulate excavations and the art market were launched, while the broader legal constructs gradually progressed from a punitive system towards a preventive one. This means that the initial harsh punishments based on strappado and the loss of employment were replaced with time by methods that tended to inhibit and preclude misdemeanours. The procedures related to the licences’ approval followed this progression too. In 1750, with the *Edict Valenti Gonzaga*, the supervision on diggings and sales became more rigorous and thorough – although not flawless. Within the new executive structure, the assessors for paintings, sculptures and other antiquities were expected to carry out full inspections on the artefacts, and prepare an advising report about their quality and conservation. Following this review, the commissary was entitled to approve or reject a licence, which needed to be countersigned by the camerlengo as well. Although infringements were not entirely eradicated, it is certain that the tightening of inspections and the growing efforts to prevent violations managed to reduce both illegal outflows and opportunities to remove the artefacts illegally.

A further instrument constituting a strong tool of prevention was the catalogue of artefacts, the register listing the ‘important objects’ worthy of protection within each jurisdiction. In this case, the early models of state inventory are not to be found in the Papal States. To identify the origins of such a legal instrument we need to turn our attention to Sweden and the Republic of Venice, specifically between the late 1600s and the early 1700s ^63^. The popes, for their part, did not establish any form of record of the local artefacts before 1802, with the so-called *Edict Doria Pamphilj* ^64^. However, as these aspects can be considered part of a different historical and chronological narrative, we will redirect our focus to the early modern legislation flourished in Europe after the example of Rome, suggesting further literature in the footnotes.

## Conclusion

The role of the papal model in inspiring the formation of local systems of heritage protection in the Old Italian States was assessed soon after the unification of Italy in 1861 ^65^. Further analysis has been offered by recent Italian scholarship connecting both juridical and artistic viewpoints, among which the studies of Emiliani of 1974 and Speroni of 1988 need to be remarked ^66^. Nonetheless, it is not difficult to understand that the legal paradigms experimented in Rome had a crucial function also in the increased attention on the heritage safeguarding in other European States. Indeed, if we accept that the papal examples were adopted in various areas of the Italian peninsula – tailored and adapted to the local needs – it is also likely that these were integrated within the domestic systems of other countries too.

The supremacy assigned to the legal traditions of both Roman law and Canon law in Europe offers a valid support in this perspective ^67^. Together with the large circulation of the *Corpus iuris civilis* and the *Corpus iuris canonici*, since the late Middle Ages increasing quantities of scholars and students converged to the universities in the centre-north Italy from all over the continent to study law ^68^. After a long training in the juridical disciplines, the graduates obtained a *status* to practice the legal professions in their homelands. In particular, it is known that the students belonging to the so-called *Natio Germanorum* – the community of the expats from north-east Europe – were extremely numerous in the universities of Bologna and Padua ^69^. Despite the fact that no class was explicitly devoted to the policies on the protection of antiquities, it cannot be excluded that knowledge on this form of legislation was also disseminated. Before the popes started issuing edicts on this matter, indeed interests on the preservation of old buildings had been shown by Roman emperors, such as Theodosius and Majorian ^70^; this was surely covered by the academic discussions on Roman and Canon law.

Yet, apart from specific juridical scholarship, it is easy to suppose that the benefits deriving from the custody of the ‘local glories’ were evident to many monarchs and governors across Europe. As already mentioned regarding the popes, the heritage protection brought advantages not only to the society as a whole, but also above all to the sovereigns seeking to legitimise their authority and power. The first governments working in this direction were in the Kingdom of Castilla and the Grand Duchy of Tuscany, where instructions on the safeguard of local remains were published in the 1570s. The rulers’ interest in the symbolic significance of antiquities substantiated, in both cases, the protection of individual monuments, inscriptions, and coats of arms which highlighted the past of the respective country ^71^. In the seventeenth century, the authorities of Denmark and Sweden demonstrated a parallel concern when issuing rules to preserve the ancient runic inscriptions in their relevant kingdoms. The legitimation of the kings’ power passed through the recognition of the mythic and outstanding history of their people; the protection of such evidence, therefore, was required to construct their dynastic hegemony, which was to be affirmed within the state and the entirety of Europe ^72^. Sweden, as mentioned above, was also one of the first countries to found a register of the ‘important materials’ to protect in 1666: it is not surprising that, at the outset, these consisted essentially of runic inscriptions.

The spread of the Enlightenment in the eighteenth century would bring about new vigorous perceptions on the importance of protecting the heritage, which were informed by cultural, juridical, artistic, and philosophical innovations all over Europe. In this context, several more countries followed the model of the Papal States, adjusting specific prescriptions to the features and the needs of the local traditions: Portugal, the Netherlands, the Kingdom of Naples, the Duchy of Parma, the Austrian Empire, some regions in the German areas, and possibly even more ^73^. Before concluding, a final significant aspect that emerged from the concepts on the heritage protection after the Enlightenment should be observed, concerning the functions of two conflicting tendencies in eighteenth-century culture and society. I refer to the ‘cosmopolitanism’ of the scholars involved in the debates within the Republic of Letters across Europe, and the ‘territorialism’ implied in the urgency to preserve ancient relics, monuments, and paintings within the pertinent country. In the perspective of this research, it can be affirmed that such an inconsistency substantiated the origin of the contemporary practices on heritage safeguard. Currently, the material works of the past are largely considered worldwide treasures that need to be protected for the advancement of universal culture and knowledge. On the other hand, no one would disagree that these should be retained in the place where they were originally created, or where they have been transferred after a legal exchange. This is one of the most prominent elements that – among others – I think should be maintained from these early-modern edicts.

## Data availability

All data underlying the results are available as part of the article and no additional source data are required.

## Footnotes

^1^ This article derives from a brief excerpt that is included in my forthcoming monograph: Mannoni, 2021a. In the past few months I have expanded, improved and enriched the initial research offered there. However, due to the long-term schedule of the publishing house, this article will be available to the readers before the monograph.

^2^ “Difficile chiedere ai secoli della creatività una riflessione così profondamente storicista qual è quella che fa nascere le categorie e ne definisce parallelamente l’equivalente verbale”. Emiliani, 1974, 36. Translation completed by the author.

^3^ In this article, only the most important edicts are discussed. Further perspectives on the laws issued in the Papal States between the early 1400s and late 1700s are in Condemi, 1987; Curzi, 2004; Emiliani, 2015; Mannoni, 2021a; Speroni, 1988.

^4^ “Civibus”; “Viarum, stratarum, platearum”. Tomassetti, 1857–72, vol. 4, 716–718. Translations of the laws from Latin completed by Alessandro Fiandra.

^5^ Bianca, 2008.

^6^ “Locorum tam publicorum quam privatorum”; “aedificiorum lapideorum et ligneorum, parictum tignorum, banchorum, tectorum, tabulatuum, […] apothecarum et pontium, portarum, passorum, acquarum decuursum, canalium et meatuum, necnon urbanorum et rusticorum praediorum, pratorum”. Tomassetti, 1857–72, vol. 4, 716–718.

^7^ “Basilicae, ac Ecclesiae ejusdem Urbis, & pia, ac religiosa loca, in quibus plurimae Sanctorum reliquiae resident”. Fea, 1802, 82–84.

^8^ “Demoliri, destruere, seu comminuere, aut rumpere, seu in calcem convertere”. Ibid.

^9^ “Porphyreticos, marmoreos, et alios diversorum generum, et colorum lapides”. Fea, 1802, 84–86.

^10^ “Antiqui marmoris, et saxi, literis, monumentisque incisi”. This was a letter of appointment. Fea, 1802, 91.

^11^ Regarding this crucial duality in Raphael’s mission as prefect see the pamphlet of Settis, 2020.

^12^ “Damnabilius”; “statuas, signa, tabulas, marmoreas, atque aeneas, porphyreticos, et numidicos, aliorumque generum lapides”; “extra Urbem in alienas Terras, ac Civitates asportari”. Edict with no title. Fea, 1802, 94–96.

^13^ A major record of the people appointed as commissary of antiquity between 1534 and 1836 is in Ridley, 1992.

^14^ The edict (with no title) mentioned Marco Antonio Amulio and Alfonso Gesualdi as prefect and conservator respectively, but Ridley, 1992 do not record any data on them. Highly likely, both posts were ineffective.

^15^ “Antiqua marmora, elaborata, et simplicia, statua, capita, metalla, gemmas, numismata, vasa, et poculas aenea, argentea, aurea, tabulas, et inscriptiones”. Fea, 1802, 99–102.

^16^ “Eaque ut antiqua vendere”. Ibid.

^17^ Tomassetti, 1857–72, vol. 8, 88–95.

^18^ The development of the concepts of private and public property concerning the heritage is analysed in Valsecchi & de’ Prati, 2009.

^19^ Prominent studies in this field are by Rizzo, see for instance Rizzo, 1986 and Rizzo, 2004.

^20^ Schioppa, 2016.

^21^ Ridley, 1992.

^22^ Schioppa, 2016 offers examples of other areas of law that encompassed the same change of approach.

^23^ “Figure, Statue, Antichità, Ornamenti, o lavori antichi come moderni, di marmo, metallo, o d’altre pietre di qualsivoglia sorte, etiam in pezzi”. *Edict Aldobrandini – Prohibitione sopra l’estrattione di Statue di marmo o di metallo, Figure, Antichità e simili,* issued under Pope Urban VIII. Emiliani, 2015, 35–36. Translations of the laws from early modern Italian completed by Amedeo Ceresa Genet.

^24^ The strappado, or corda, or reverse hanging, was a system of torture. The hands of the victims were tied behind their back, and they were suspended by a rope attached to the wrists.

^25^ “Statue, figure, bassi rilievi, colonne, vasi, alabastri, agate, diaspri, amatisti, o altri marmi, gioie, e pietre lavorate, e non lavorate, torsi, teste, fragmenti, pili, piedestalli, inscrittioni, o altri ornamenti, fregi, medaglie, camei, o intagli di qualsivoglia pietra, overo metallo, oro, argento di qualsivoglia materia antica, o moderna, nè meno figure, o pitture antiche, o altre opere in qualsivoglia cosa sculpite, depinte, intagliate, commesse, lavorate [...]”. *Edict Sforza – Editto sopra l’estrattioni, e cave di Statue, Figure, Intagli, Medaglie, Inscrittioni di marmo, di mischio, metallo, Oro, Argento, Gioie, e cose simili antiche e moderne,* issued under Pope Innocent X. Emiliani, 2015, 36–41.

^26^ *Edict Altieri – Prohibitione sopra l’estrattione di Statue di marmo o metallo, Figure, Antichità e simili* issued under pope Innocent XI. First *Edict Spinola – Prohibitione sopra l’estrattione di Statue di marmo, o metallo, Figure, Antichità e simili* issued under Pope Clement XI. Emiliani, 2015, 41–46.

^27^ Haskell, 1963.

^28^ To understand the prolific exchanges of scholars, artists, knowledge and research methods in eighteenth-century Europe, see Pinelli, 2009. See also Brook & Curzi, 2010.

^29^ Fumaroli, 2007 and Fumaroli, 2018; Mori & Veca, 2019; Piva, 2014b; Susinno, 2000.

^30^ There is extensive literature available on this topic. I suggest: Avery-Quash & Huemer, 2019; Bignamini & Wilton, 1996; Bignamini & Hornsby, 2010; Black, 1992 and Black, 2003; Coen, 2018; Hersant, 2001; Piva, 2012.

^31^ Second *Edict Spinola – Editto sopra le Pitture, Stucchi, Mosaici, et altre Antichità, che si trovano nelle cave, Inscrizioni antiche, Scritture, e Libri manoscritti,* issued under Pope Clement XI. Emiliani, 2015, 46–49.

^32^ “Libri scritti a mano tanto Volgari, e Latini, quanto Greci, Ebraici […] divisi, rotti, e sciolti, come pure Instromenti, Processi, Inventarij, Lettere, Bolle, Brevi, Diplomi, e qualunque altra sorte di carte”. In the Grand Duchy of Tuscany an early form of protection for the ‘ancient written memories’ was established in 1606. Ibid.

^33^ Third *Edict Spinola* on documents and archives, issued under pope Clement XI. Mentioned without its full title in Speroni, 1988, 18–20.

^34^ “Stranieri, e sudditi d’altri Principi, […] ogni volta che apparisca […] dimora in Roma per lo spazio di un mese”. Fourth *Edict Spinola - Prohibizione sopra l’estrazione di Statue di marmo, o metallo, Figure, Antichità e simili*, issued under Pope Clement XI. Emiliani, 2015, 49–50.

^35^ Black, 1992 and Black, 2003; Beltrán *et al.*, 2001.

^36^ First *Edict Albani – Editto sopra li scalpellini, segatori di marmi, cavatori et altri,* issued under Pope Benedict XIII. Emiliani, 2015, 50–51.

^37^ “Statue, o frammenti di esse, Torsi, Teste, Bassi Rilievi, Piedistalli, Colonne, Capitelli, Inscrizioni, Vasi, Urne, ed altri ornamenti [...]”. Ibid.

^38^ Second *Edict Albani – Prohibizione dell’estrazione delle Statue di marmo, o metallo, Pitture, Antichità e simili,* issued under Pope Clemente XII. Emiliani, 2015, 52–55.

^39^ “Cose […] alterate e falsificate”. Ibid.

^40^ “Pitture, Mosaici, e Quadri, e altre simili opere tanto antiche, quanto moderne”. Ibid.

^41^ Condemi, 1987; Haskell, 1963.

^42^ For a wider analysis of the market of paintings in Rome in 1700s, see Coen, 2010.

^43^ It is noteworthy that the Grand Duchy of Tuscany issued a regulation to protect modern paintings in 1602. However, only the works of 19 selected Renaissance artists were banned from export at this stage. See Speroni, 1988.

^44^ *Edict Valenti Gonzaga – Proibizione della estrazione delle Statue di marmo, o metallo, Pitture, Antichità e Simili,* issued under Pope Benedict XIV. Emiliani, 2015, 56–64. See also Condemi, 1987; Speroni, 1988.

^45^ The edicts issued in Rome at the beginning of 1800s were sharper and more comprehensive in their conceptual, legal and administrative constructs than the edict of 1750, but their analysis falls out the scope of this essay. I suggest: Curzi, 2004; Mannoni, 2021a; Pinelli, 1978–79.

^46^ The edicts issued in 1600s dealt with illegal excavations and smuggling altogether; in the early 1700s these issues were regulated within separate protocols. The edict of 1750 considered the two misdemeanors jointly, offering improved and effective solutions compared to the regulations of 1600s.

^47^ Schnapp, 1996.

^48^ In 1755, the Bourbons explicitly cited the example of the Papal States in the introduction of their first *Prammatica* on the protection of Neapolitan artefacts. D’Alconzo, 1999; Speroni, 1988.

^49^ Illuminismo giuridico. Schioppa, 2016.

^50^ Fattori, 2011; Rosa, 2000.

^51^ Schioppa, 2016. See also Romagnani, 2007.

^52^ “Statue, Figure, Bassirilievi, Colonne, Vasi, Alabastri, Agate, Diaspri, Amatiste, ed altri marmi preziosi, Gioie, e Pietre lavorate, Dorsi, Teste, Frammenti, Pili, Piedestalli, Inscrizioni, o altri Ornamenti, Fregi, Medaglie, Camei, Corniole, Monete, o Intagli di qualsivoglia Pietra, ovvero Metallo, Oro, Argento di qualsivoglia materia antica, o moderna, né meno Figure, Quadri, Pitture antiche, o altre opere in qualsivoglia cose sculpite, e depinte, intagliate, commesse, lavorate, o in altro modo fatte […] esistenti in Roma, e fuori Roma”. Emiliani, 2015, 56–64.

^53^ The Capitoline Museum is traditionally indicated as the oldest museum in the world. In 1471 Pope Sixtus V donated a group of ancient bronzes to the citizens of Rome; these were placed in the buildings on the Capitoline hill, later converted into the Capitoline Museum.

^54^ Liverani, 2000; Brook & Curzi, 2010. There is extensive literature on the foundation of the Capitoline and Vatican museums in eighteenth-century Rome, here I suggest the publications of Arata and Pietrangeli, in particular: Arata, 2016; Pietrangeli, 1985.

^55^ “Pittura”, “Scultura”, Camei, Medaglie, Incisioni, ed ogni altra sorte di Antichità”. Emiliani, 2015, 56–64.

^56^ Ridley, 1992.

^57^ Publications on Wincklemann are countless. For the perspectives of this essay, I recommend: Piva, 2014b; Pommier, 2003; Pinelli, 2010.

^58^ *Edict Sforza – Editto sopra l’estrattioni, e cave di Statue, Figure, Intagli, Medaglie, Inscrittioni di marmo, di mischio, metallo, Oro, Argento, Gioie, e cose simili antiche e moderne,* issued under Pope Innocent X. Emiliani, 2015, 36–41.

^59^ *Edict Valenti Gonzaga – Proibizione della estrazione delle Statue di marmo, o metallo, Pitture, Antichità e Simili,* issued under Pope Benedict XIV. Emiliani, 2015, 56–64.

^60^ Edict with no title, issued by Pope Paul III. Fea, 1802, 94–96.

^61^ “A fede della qualità, quantità […] delle cose suddette”. Emiliani, 2015, 36–41.

^62^ Fourth *Edict Spinola - Prohibizione sopra l’estrazione di Statue di marmo, o metallo, Figure, Antichità e simili*, issued under Pope Clement XI. Emiliani, 2015, 49–50.

^63^ Mannoni, 2021b; Piva, 2014a; Speroni, 1988.

^64^ Curzi, 2004; Mannoni, 2021a; Pinelli, 1978–79.

^65^ The Papal States were annexed to Italy in 1870. See, for instance, the volumes on law of Fiorelli, 1881; Mariotti, 1892.

^66^ Emiliani, 1974; Speroni, 1988.

^67^ Essential studies on Roman law and Canon law starts with Le Bras, 1976 and Vinogradoff, 1909.

^68^ Interesting perspectives on the circulation of juridical knowledge in early modern Europe are offered in Bellomo, 1995; Kelley, 1990; Schioppa, 2016.

^69^ Forlivesi, 2008.

^70^ As emerges in some constitutions included in the *Codex Theodosianus* of 438, and in the *Novella Maiorani 4 – De aedificiis publicis* of 458. Angeletti, 2019; Pharr, 1952.

^71^ Emiliani, 2015; Lopez-Trujillo, 2006; Speroni, 1988.

^72^ Adlercreutz, 2017; Mannoni, 2021b; Schnapp, 1996.

^73^ I am currently working on a comparative analysis of the laws issued in these countries in the 1700s; my aim is to divulge it as soon as the results are satisfactory. From this list I have deliberately omitted the Republic of Venice, as it followed an individual path in elaborating quite particular regulations on the conservation of the local artefacts.
