# Supplementary material for: Protecting antiquities in early modern Rome: the papal edicts as paradigms for the heritage safeguard in Europe
Source: Open Res Eur. 2021 May 13;1:48. [Version 1] doi: 10.12688/openreseurope.13539.1 (PMC10445861; doi:10.12688/openreseurope.13539.1)
Supplement: Peer review history

# Peer review: Reviewer response for version 1

**DOI:** 10.21956/openreseurope.14609.r26890

**Published:** 2021-06-21

**Authors:** Stefania Ventra

**Affiliations:** 1 Archivio del Moderno, Accademia di Architettura, Università della Svizzera Italiana, Balerna, Switzerland

This article deals with the legislation on the protection of the archaeological and artistic heritage developed in the Papal States between the 15th and the 18th Century, proposing to acknowledge it as a paradigm that inspired the heritage safeguard in Modern Europe.

 The protection of the heritage in the Papal States was at first linked to antiquity and in particular to architecture, that is, to real estate. Starting from the sixteenth century, the article follows the increasingly specific and extensive insertion of movable objects to protection, and above all of modern heritage from 1624 (Edict Aldobrandini) onwards.

 Acutely, the author considers the legislative outcomes  against the occurrences in the history of culture, taste and collecting in the respective moments; at the same time, she also focuses on much less common issues in the studies on this subject, such as the reception of legislation. One of the strong points of the reasoning is, in fact, the consideration of the language used in the edicts, that is Latin, as a limit to the understanding, dissemination and compliance with the regulations. Rightly, the laws on the protection of the heritage are also analyzed in the context of the broader legislation issued in  the various historical moments, and although the text summarizes the contents of a monograph in some parts, this always meets the elements useful to follow the reasoning.

 The chronological progression finally reaches the eighteenth century and the role of Cardinal Silvio Valenti Gonzaga in the pontificate of Benedict XIV. These virtuous figures, indeed, operated in a favorable philosophical, religious and cultural context in general. The heritage protection regards now all kind of objects (also modern pictures, sculptures, mosaics, and so on).  In this regards, it should be emphasized that the attention to modern works goes hand in hand with the establishment of the social history of the artists, and the recognition of painting and sculpture as liberal arts.

 Rome in the mid-Eighteenth century combines improved legislation, museums, archaeological excavations, erudition, as well as increased presence of administrative offices. All these factors will produce the State apparatus for the protection of the artistic heritage that will constitute a model for the whole of the Enlightened Europe, according to an updated concept that aims to merge prevention as well as punishment.

If any, are all the source data and materials underlying the results available?

Yes

Is the work original in terms of material and argument?

Partly

Is the work clearly and cogently presented?

Yes

Is the argument persuasive and supported by evidence?

Yes

Does the research article contribute to the cultural, historical, social understanding of the field?

Yes

Does it sufficiently engage with relevant methodologies and secondary literature on the topic?

Yes

Reviewer Expertise:

Art History; History of conservation and restoration of cultural heritage

I confirm that I have read this submission and believe that I have an appropriate level of expertise to confirm that it is of an acceptable scientific standard.

# Peer review: Reviewer response for version 1

**DOI:** 10.21956/openreseurope.14609.r26891

**Published:** 2021-06-01

**Authors:** Madalena Costa Lima

**Affiliations:** 1 ARTIS – Instituto de História da Arte da Faculdade de Letras, Universidade de Lisboa, Lisboa, Portugal; 2 CLEPUL – Centro de Literaturas e Culturas Lusófonas e Europeias da Faculdade de Letras, Universidade de Lisboa, Lisboa, Portugal

The thesis proposed in this paper is presented in an original way, regarding the arguments, or the use of these arguments, for which the author, C. Mannoni, is to be congratulated. Although the work fills important gaps in heritage and cultural studies areas – specially considering the eighteenth century –, and so in the history of art, the century-old Italian legislation related to heritage, in which the papal edicts are included, has been published and highlighted as relevant sources by several historians, or researchers on art and heritage studies field. The most notable of these authors is possibly A. Emilliani, the first to be quoted in the paper, and properly referred to some other times along the text. Indeed, we owe him the most important compilation of the legislation on “artistic and cultural and objects” (“beni artistici e culturali”) produced in the Italian states between the sixteenth and nineteenth centuries, a work published with an “Introduzione” by Emilliani, whose interpretation Mannoni follows closely.

 The juridical sources related to Italy are carefully chosen and correctly used to serve the thesis presented in the paper. However, the idea that the papal legislation was a “paradigm” for “Europe” would benefit with a few more lines dedicated to the situation in other European states, as they are fundamental to support the thesis, and are only denoted, or properly articulated, in the “Conclusion” of the work. Probably, the work would profit from the use of other than juridical sources, such as letters, literature or historiographical sources, which demands a deeper longer research, perhaps not required to the length or scope of a paper. The same is valid for the secondary literature referred in the work. For instance, a Portuguese researcher has suggested that the Portuguese law on “ancient monuments” from 1721 was inspired by the Roman legislation (Paulo Oliveira Ramos, “O alvará régio de 20 de agosto de 1721 […]).

 I find it fundamental to consider Aloïs Riegl’s *Der modern Denkmalkultus: sein wesen und seine Entstehung* (“The modern cult of the Monuments: its character and origin”), the first systematic analysis of heritage values, a seminal work, published in 1903, which is still the most methodical, and possibly the most important, text on the notions of heritage, as Françoise Choay shows in her *L’allegorie du patrimoine*, a famous essay on heritage awareness, many times edited and translated, that would also enriched Mannoni’s paper. The references of Riegl and Choay are essential to clarify the concept of “heritage”, “historical and artistic heritage”, the key concept of the paper. Indeed, this basic, central notion, a modern notion that definitely emerged in Europe during the eighteenth century, is not suitably clarified in the paper, a text where the historical value is perhaps never plainly highlighted, and tends to be mixed with the artistic value. The legislative authorities were first alert and motivated with/by the memory values of the objects – “ *marmorum et lapidum”*, “slabs”, “stones”, “marbles, both raw and worked”, as designated in the sources, were primarily esteemed as ancient artifacts. As so, the author may consider refining assertions such as “Conservation results from the identification of an object as an artwork”, with a view of enhancing the scientific robustness that the paper undeniably merits, as it gives a new, more enlarged reading of the papal edits, and fills a gap in the historiography.

If any, are all the source data and materials underlying the results available?

Yes

Is the work original in terms of material and argument?

Partly

Is the work clearly and cogently presented?

Yes

Is the argument persuasive and supported by evidence?

Partly

Does the research article contribute to the cultural, historical, social understanding of the field?

Yes

Does it sufficiently engage with relevant methodologies and secondary literature on the topic?

Partly

Reviewer Expertise:

Heritage and Theory of Restoration; Enlightenment culture; History of Art.

I confirm that I have read this submission and believe that I have an appropriate level of expertise to confirm that it is of an acceptable scientific standard.
